# *Lactobacillus casei* expressing Internalins A and B reduces *Listeria monocytogenes* interaction with Caco‐2 cells *in vitro*

**DOI:** 10.1111/1751-7915.13407

**Published:** 2019-04-15

**Authors:** Moloko G. Mathipa, Mapitsi S. Thantsha, Arun K. Bhunia

**Affiliations:** ^1^ Department of Biochemistry, Genetics, and Microbiology University of Pretoria Pretoria South Africa; ^2^ Molecular Food Microbiology Laboratory Department of Food Science Purdue University West Lafayette IN USA; ^3^ Department of Comparative Pathobiology Purdue University West Lafayette IN USA; ^4^ Purdue Institute of Inflammation, Immunology, and Infectious Disease Purdue University West Lafayette IN USA

## Abstract

*Listeria monocytogenes* has been implicated in a number of outbreaks including the recent largest outbreak in South Africa. Current methods for prevention of foodborne *L. monocytogenes* infection are inadequate, thus raising a need for an alternative strategy. Probiotic bioengineering is considered a prevailing approach to enhance the efficacy of probiotics for targeted control of pathogens. Here, the ability of *Lactobacillus casei* expressing the *L. monocytogenes* invasion proteins Internalins A and B (*inlAB*) to prevent infection was investigated. The *inlAB* operon was cloned and surface‐expressed on *L. casei* resulting in a recombinant strain, Lbc^Inl^ ^AB^, and subsequently, its ability to inhibit adhesion, invasion and translocation of *L. monocytogenes* through enterocyte‐like Caco‐2 cells was examined. Cell surface expression of InlAB on the Lbc^Inl^ ^AB^ was confirmed by Western blotting and immunofluorescence staining. The Lbc^Inl^ ^AB^ strain showed significantly higher (*P *<* *0.0001) adherence, invasion and translocation of Caco‐2 cells than the wild‐type *L*. *casei* strain (Lbc^WT^), as well as reduced *L. monocytogenes* adhesion, invasion and transcellular passage through the cell monolayer than Lbc^WT^. Furthermore, pre‐exposure of Caco‐2 cells to Lbc^Inl^ ^AB^ significantly reduced *L. monocytogenes*‐induced cell cytotoxicity and epithelial barrier dysfunction. These results suggest that InlAB‐expressing *L*. *casei* could be a potential practical approach for prevention of listeriosis.

## Introduction

*Listeria monocytogenes* is a Gram‐positive, facultative intracellular foodborne pathogen that persists in the diverse environment within and outside mammalian host cells (Vazquez‐Boland *et al*., 2001; Czuprynski, 2005). The severity of the disease listeriosis depends on the host immune status. The infection in immunocompetent individuals is commonly self‐limiting febrile gastroenteritis, while it results in meningitis and encephalitis in immunocompromised individuals. In expectant women, it can spread to the uterus, thereby affecting the fetus, consequently causing complications such as spontaneous abortions, stillbirths or premature births (Schuchat *et al*., 1991; Wolfe *et al*., 2017). Incidences of listeriosis are much lower than diseases caused by most foodborne pathogens; however, its high case fatality rate (20–30%) has made it a considerable public health concern (Scallan *et al*., 2011; de Noordhout *et al*., 2014). The largest listeriosis outbreak ever recorded is the recent one reported in South Africa (2017–2018), linked to consumption of ready‐to‐eat (RTE) sausage called Polony. It resulted in a total of 1060 cases, of which 216 were fatal (Allam *et al*., 2018) (http://www.nicd.ac.za/index.php/listeriosis-outbreak-situation-report-_4july2018/).

As an intracellular pathogen, *L. monocytogenes* can invade non‐phagocytic cells and cross the intestinal (Nikitas *et al*., 2011; Drolia *et al*., 2018), blood–brain (Ghosh *et al*., 2018) and feto‐placental (Robbins *et al*., 2010; Wolfe *et al*., 2017) barriers. It attaches to and enters into mammalian cells, evades destruction by host phagocytic cells, multiples intracellularly and then spreads to adjacent cells (Radoshevich and Cossart, 2018). Virulence factors responsible for its adhesion include but are not limited to *Listeria* adhesion protein (LAP), autolysin amidase (AmiA) and the Internalin (Inl) family of proteins (InlA, InlB, InlJ and InlF) (Camejo *et al*., 2011; Radoshevich and Cossart, 2018). *Listeria* adhesion protein is an alcohol acetaldehyde dehydrogenase (*lmo1634*) that promotes adhesion of *Listeria* during the intestinal infection phase (Pandiripally *et al*., 1999; Jagadeesan *et al*., 2010; Bailey *et al*., 2017). It interacts with the epithelial receptor, heat‐shock protein 60 (Hsp60) (Wampler *et al*., 2004; Jagadeesan *et al*., 2011), and activates NF‐κB and myosin light chain kinase (MLCK) resulting in mislocalization of tight junction proteins and opening of the cell–cell junction for bacterial passage into the lamina propria (Drolia *et al*., 2018).

For host cell invasion, the pathogen uses InlA and InlB (Robbins *et al*., 2010; Stavru *et al*., 2011), which binds to the host cell receptor E‐cadherin (Mengaud *et al*., 1996) and the hepatocyte growth factor receptor c‐Met (Shen *et al*., 2000) respectively. InlA also aids crossing of the gut epithelial barrier by transcytosis (Nikitas *et al*., 2011), while InlB facilitates the invasion of human hepatic and M cells (Chiba *et al*., 2011; Disson and Lecuit, 2013). InlA and InlB are secreted proteins (Trost *et al*., 2005) and remain covalently attached to the peptidoglycan via LPXTG motif and teichoic acid via GW motif of *L*. *monocytogenes* cell wall respectively (Braun *et al*., 1997; Schubert *et al*., 2002). The bacterium then employs listeriolysin O (LLO) and phospholipases (PlcA and PlcB) to escape from the vacuoles and actin polymerization protein (ActA) to move from cell to cell (Portnoy *et al*., 1992; Camejo *et al*., 2011). There is currently no vaccine for this pathogen. Only precautionary guidance stated by the Centers for Disease Control and Prevention summarizes the importance of hygiene during food preparation and handling, as well as avoidance of certain RTE foods by high‐risk groups.

Probiotics have been used to restore the balance of the gut microbial ecosystem and for control of pathogenic infections. They prevent or control foodborne illnesses through competitive exclusion of pathogens, stimulation of the host immune system and tightening of the gut barrier (Amalaradjou and Bhunia, 2012; Behnsen *et al*., 2013). Several studies have reported their use to combat *L*. *monocytogenes* (Touré *et al*., 2003; Corr *et al*., 2007; Aguilar *et al*., 2011). Despite the proven success of probiotics for control of enteric pathogens, they are not without shortcomings. Their disadvantages are that their action is non‐specific in nature, they sometimes fail to block attachment of some pathogens to their specific receptors, and in certain instances, they induce low levels of an immune response (Bauer *et al*., 2002; McCarthy *et al*., 2003; Koo *et al*., 2012). Novel probiotic strains with enhanced desirable attributes can be designed by considering these limitations of traditional probiotics, as well as the behaviour and disease processes of the pathogens (O'Toole *et al*., 2017; do Carmo *et al*., 2018). These novel strains that can prevent pathogenic infections, deliver drugs or vaccines, mimic surface receptors and enhance host immune responses are developed using genetic modification (Steidler, 2003; Buccato *et al*., 2006; Kajikawa *et al*., 2007; Wells and Mercenier, 2008; Unnikrishnan *et al*., 2012; Amalaradjou and Bhunia, 2013; Ryan and Bhunia, 2017).

A recombinant *Lactobacillus paracasei* strain expressing the LAP of *L. monocytogenes* (Lbp^LAP^) was previously developed in our laboratory, and it showed enhanced inhibition of *L. monocytogenes* interaction with Caco‐2 cells when compared to its wild‐type counterpart (Koo *et al*., 2012). Recently, this same gene was cloned and expressed into *L. casei* ATCC344 strain and the resultant recombinant strain Lbc^LAP^ exhibited a similar anti‐*listeria* effect (unpublished). Researchers elsewhere also cloned and expressed InlA into *Lactococcus lactis* for delivering DNA intracellularly (Guimaraes *et al*., 2005; Innocentin *et al*., 2009; De Azevedo *et al*., 2015; Yano *et al*., 2018). Paradoxically, none of these studies examined whether these InlA‐expressing recombinant strains could prevent *L. monocytogenes* infection in a model system. Therefore, in the current study, our goal was to simultaneously clone and express both InlA and InlB (since both are required for cell invasion) into *L. casei*, a well‐studied probiotic strain with proven health beneficial effects (Lenoir *et al*., 2016; Jacouton *et al*., 2017), and then investigate the ability of the resultant recombinant strain to inhibit adhesion, invasion and translocation of *L. monocytogenes in vitro* in a cell culture model.

## Results

### InlAB was successfully cloned and expressed in *Lactobacillus casei*

**Figure 1:**
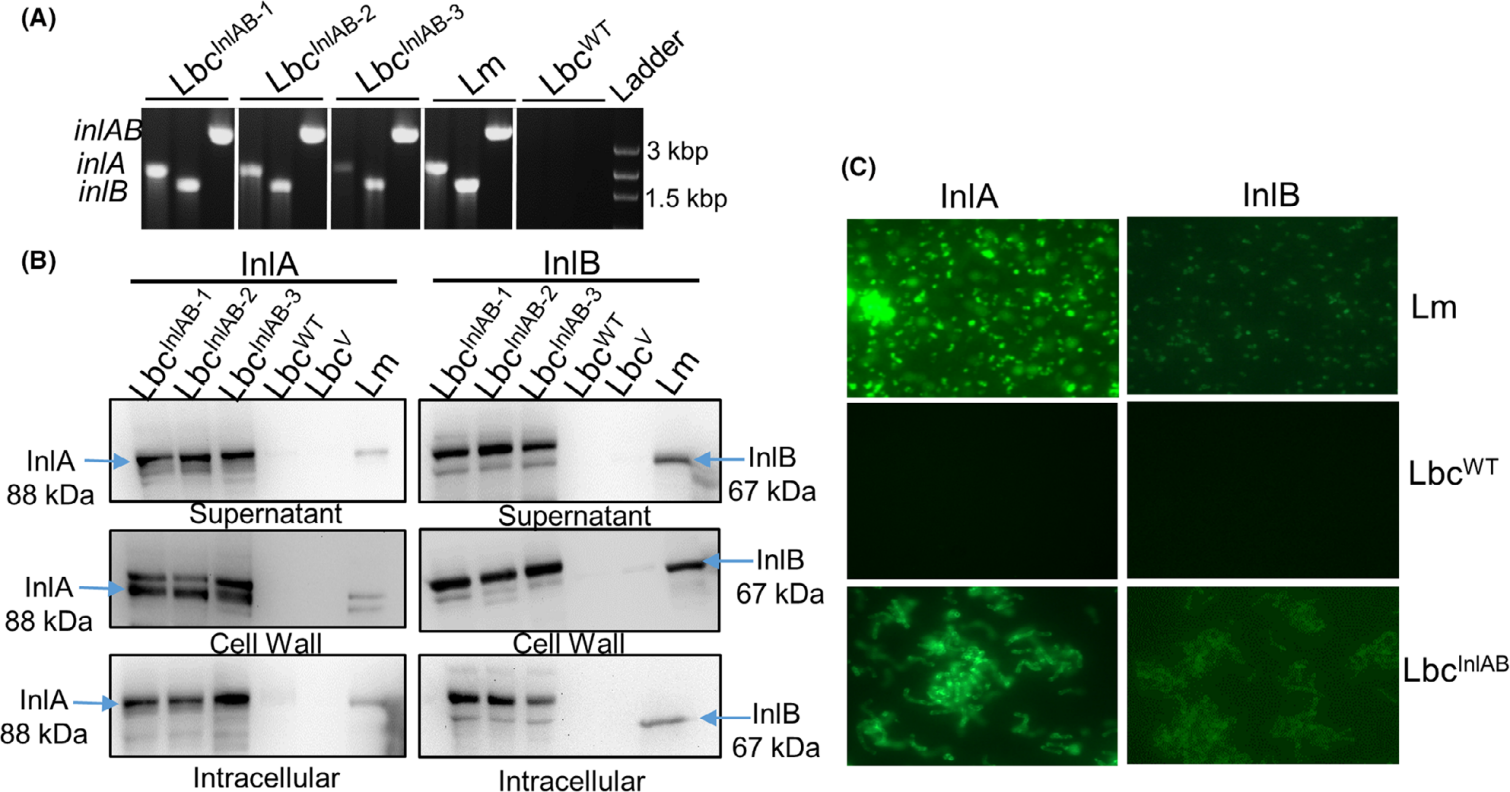
Agarose gel showing (A) PCR‐amplified gene products for *inlAB*,* inlA* and *inlB* of InlAB‐expressing 3 recombinant *Lactobacillus casei* strains (Lbc^Inl^ ^AB^ ^−1^, Lbc^Inl^ ^AB^ ^−2^, Lbc^Inl^ ^AB^ ^−3^) and *L. monocytogenes* (Lm) and Lbc^WT^. Lm: *L. monocytogenes* F4244 (positive control) and Lbc^WT^ (negative control).B. Western blot showing expression of Internalins InlA and InlB in the recombinant *L. casei* strains (Lbc^Inl^ ^AB^ ^−1^, Lbc^Inl^ ^AB^ ^−2^, Lbc^Inl^ ^AB^ ^−3^, Lbc^WT^ and Lbc^V^) in the different cellular fractions (supernatant, cell wall and intracellular) and *L. monocytogenes* F4244 (Lm). C. Immunofluorescence staining of bacteria (magnification 1000 × ) with anti‐InlA mAb‐2D12 and anti‐InlB pAb404. Lbc^Inl^ ^AB^ and Lm (control) cells indicated the presence of InlA (green) and no expression in Lbc^WT^. Anti‐InlB pAb‐404 staining produced weak signal, suggesting this antibody may not be suitable for immunofluorescence staining.

To engineer the probiotic *Lactobacillus casei* expressing *inlAB* of *L. monocytogenes*, the PCR‐amplified *inlAB* gene product and the plasmid pLP401‐T were both digested with the restriction enzymes NotI and XhoI and then subsequently ligated to produce the recombinant vector designated pLP401‐InlAB (Fig. S1A). This construct was electrotransferred into *L. casei* ATCC344 (Lbc^WT^), and three selected transformants were confirmed by PCR to contain *inlAB* operon (Fig. 1A).

Western blot assay confirmed the expression of both InlA and InlB proteins in the different cellular fractions (supernatant, cell wall and intracellular) of Lbc^InlAB^ while absent in Lbc^WT^ or Lbc^V^ (Lbc carrying only empty pLP401‐T vector) cell fractions (Fig. 1B, Fig. S1B). Immunofluorescence staining also confirmed the surface expression of InlA and InlB in Lbc^InlAB^ strain (Fig. 1C). *Listeria monocytogenes* F4244 (serotype 4b) was used as a positive control (Fig. 1B). These data indicate that both InlA and InlB were successfully expressed in Lbc^InlAB^ strain and were associated with the cell wall. Transformant 1 (Lbc^InlAB−1^) was used for the rest of the experiments.

### The InlAB expression did not affect the growth rate of Lbc^InlAB^ strain

**Figure 2:**
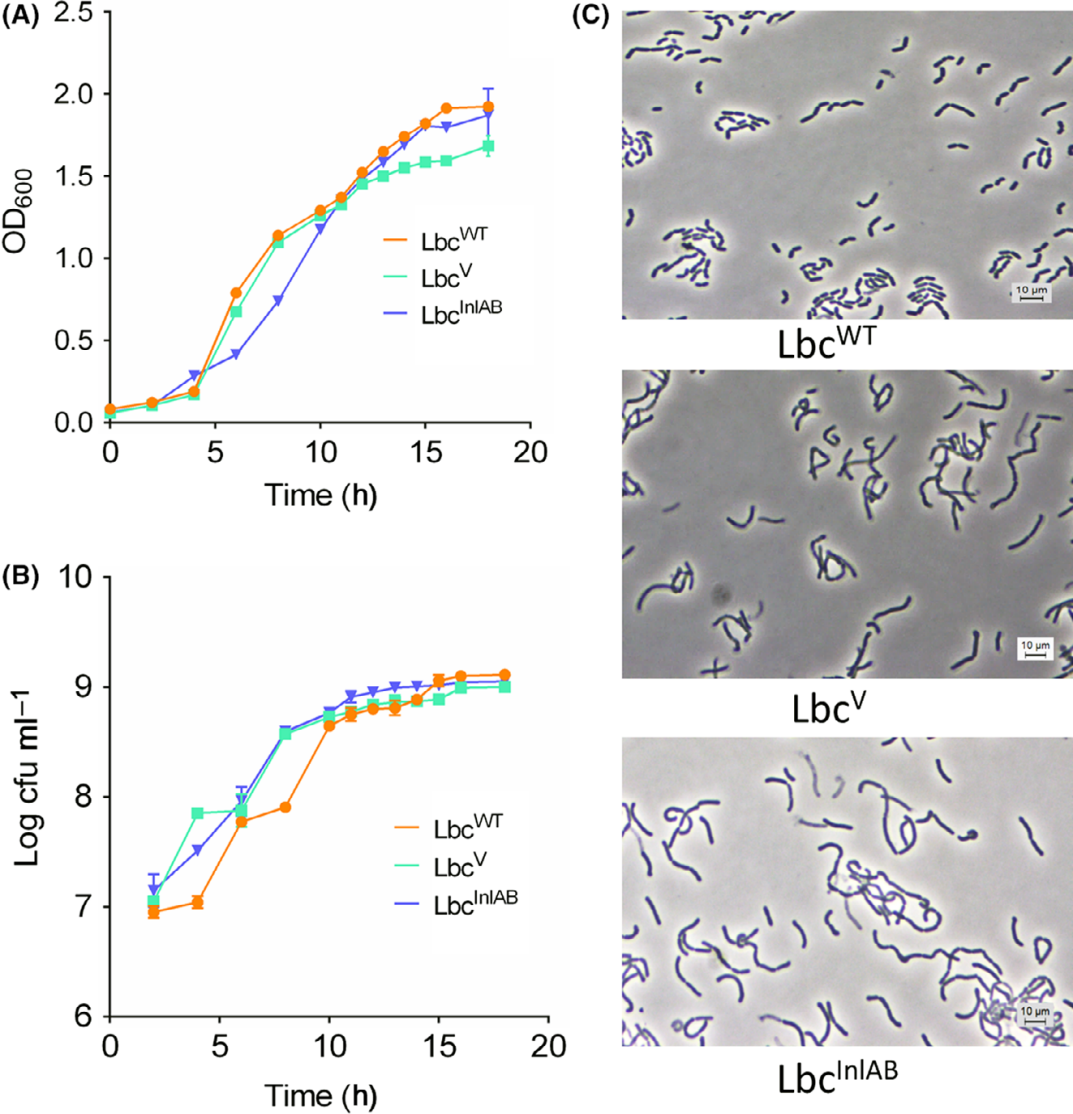
Panel showing *L. casei* growth curves. (A) Optical density measurement (OD at 600 nm), (B) bacterial counts and (C) phase‐contrast microscopic images of Lbc^WT^, Lbc^V^ and Lbc^Inl^ ^AB^. This experiment was performed twice in triplicates.

In order to determine whether the expression of InlAB affects the growth of *L. casei*, we compared growth curves of the Lbc^WT^, Lbc^V^ and Lbc^InlAB^. Both optical density (Fig. 2A) and the viable cell count (log CFU ml^−1^) (Fig. 2B) data showed similar growth profiles for all three strains over time. Furthermore, in phase‐contrast micrographs (Fig. 2C), all three strains Lbc^WT^, Lbc^V^ and Lbc^InlAB^ maintained a typical elongated curve‐shaped morphology; however, Lbc^V^ and Lbc^InlAB^ formed slightly longer chains.

### Adhesion, invasion and translocation characteristics of recombinant Lbc^InlAB^

**Figure 3:**
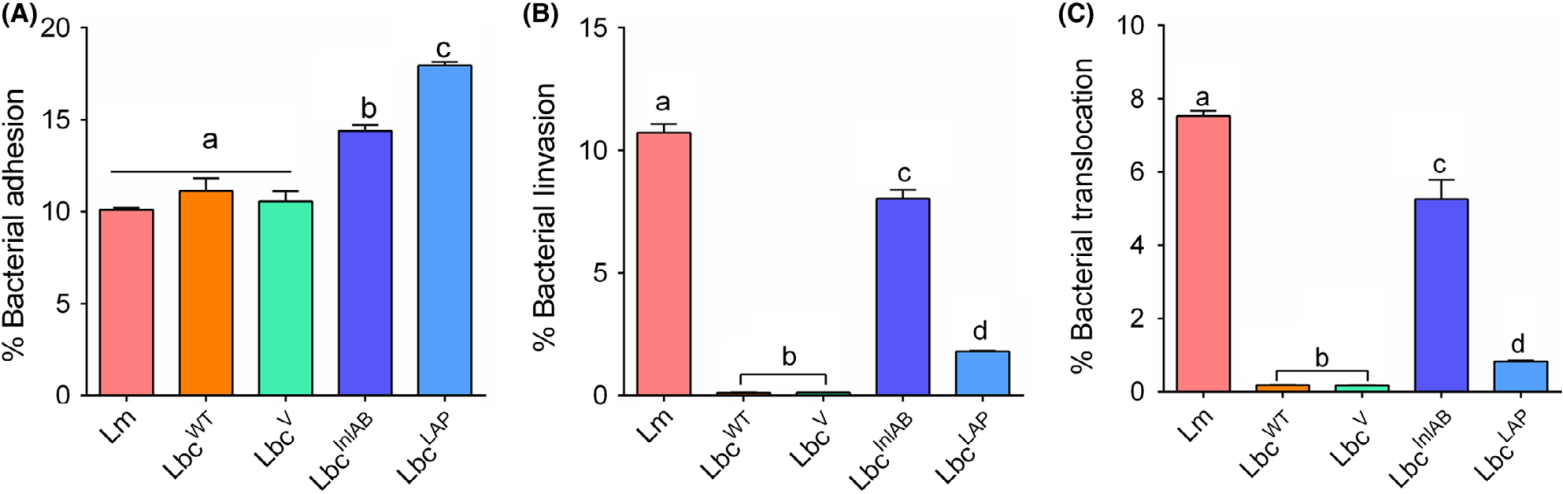
Adhesion, invasion and translocation profiles of *Listeria monocytogenes* (Lm) and *Lactobacillus casei* (Lbc) to Caco‐2 cells.A. Adhesion, (B) invasion and (C) translocation of the Caco‐2 cells by *L. monocytogenes* and *L. casei* strains (Lbc^WT^, Lbc^V^, Lbc^Inl^ ^AB^ and Lbc^LAP^). Percentages were calculated relative to the inoculums that were added to the Caco‐2 cells. Data are average (SD) of three independent experiments performed in duplicate. For each time point, bars marked with different letters (a, b, c, d) indicate significant difference at *P* < 0.05.

We compared the abilities of the *L*. *casei* strains (Lbc^WT^, Lbc^V^ and Lbc^InlAB^) to adhere to, invade and translocate through or across the Caco‐2 cells versus those of *L*. * monocytogenes* and Lbc^LAP^. Lbc^WT^ (*P* = 0.8466) and Lbc^V^ (*P* = 0.9964) showed similar adhesion profiles to Caco‐2 cells when compared to *L. monocytogenes* (Fig. 3A); however, adhesion of Lbc^InlAB^ was significantly higher than that of Lbc^WT^ (*P* = 0.0153). As expected, Lbc^LAP^ also showed higher adhesion (17.95%) than Lbc^WT^ (11.13%). These data indicate that InlAB expression augmented the ability of Lbc^InlAB^ strain to adhere to Caco‐2 cells.

In Caco‐2 cell invasion assay, Lbc^InlAB^ (8.0%) showed a significantly higher invasion (*P *<* *0.05) than the Lbc^WT^ (0.18%) or Lbc^V^ (0.13%) (Fig. 3B). *Listeria monocytogenes* as a positive control showed high invasion (10.7%). As anticipated, Lbc^LAP^ had a low invasion (0.83%), which was significantly lower (*P* < 0.0001) than that of Lbc^inlAB^.

Likewise, Lbc^InlAB^ also showed a significantly higher (*P* < 0.0001) transcellular translocation through epithelial (Caco‐2) barrier in a trans‐well set‐up than the Lbc^WT^ or Lbc^V^ strains (Fig. 3C). *L*. *monocytogenes* was able to invade and translocate across the Caco‐2 cells at significantly higher levels (*P* < 0.0001) than those obtained for all the *L*. *casei* strains. Interestingly, Lbc^LAP^ showed a significantly lower (*P* < 0.0001) paracellular translocation than the Lbc^InlAB^ strain.

### Competitive exclusion of *L. monocytogenes* by recombinant Lbc^InlAB^

**Figure 4:**
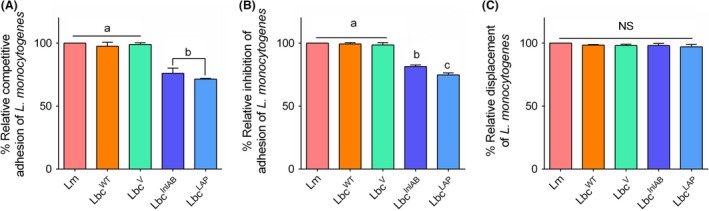
Competitive exclusion of *Listeria monocytogenes* (Lm) adhesion to Caco‐2 cells by *L. casei* strains (Lbc^WT^, Lbc^V^, Lbc^Inl^ ^AB^ and Lbc^LAP^), analysed by three different exclusion mechanisms: (A) competitive adhesion – Caco‐2 cells were exposed to *L. casei* strains with Lm simultaneously; (B) inhibition of adhesion – Caco‐2 cells were pre‐exposed to *L. casei* strains for 1 h before infection with Lm; and (C) displacement of adhesion – Caco‐2 cells were infected with Lm for 1 h before *L. casei* treatment (1 h). Adhesion of Lm alone to Caco‐2 cells was presented as 100%, and per cent adhesion was calculated relative to that. For each time point, bars marked with different letters (a, b, c) indicate significant difference at *P* < 0.05.

Probiotics inhibit pathogen colonization through the mechanism of competition, either for attachment site or for food. There are different ways by which probiotics can competitively inhibit pathogen adhesion and infection: competitive adhesion, inhibition of adhesion and displacement of adhesion (Fig. 4). Adhesion of *L. monocytogenes* to Caco‐2 cells in the absence of *L. casei* strains was recorded as 100% in all the assays and was used to calculate the relative adhesion in the presence of these strains. In the competitive adhesion assay, adhesion of *L*. *monocytogenes* was significantly reduced (*P* < 0.0001) by 24% when co‐inoculated with Lbc^InlAB^ for 1 h (Fig. 4A), while it was not reduced when it was added simultaneously with either Lbc^WT^ (*P* = 0.9136) or Lbc^V^ (*P* = 0.9986). Similar results were obtained for inhibition of adhesion assay where *L*. *casei* strains were allowed to adhere for 1 h before inoculation with *L. monocytogenes* for 1 h (Fig. 4B). Conversely, Lbc^InlAB^ failed to displace already adhered *L. monocytogenes* to Caco‐2 cells and showed no statistical differences (*P* < 0.05) when compared with Lbc^WT^ or Lbc^V^ (Fig. 4C). Interestingly, Lbc^LAP^ showed significantly higher inhibition (*P* < 0.0007) of *L. monocytogenes* than Lbc^InlAB^ (26% vs. 19%) and was unable to displace attached *L. monocytogenes* cells (Fig. 4).

### Inhibition of *L. monocytogenes* adhesion, invasion and transcellular migration over time

**Figure 5:**
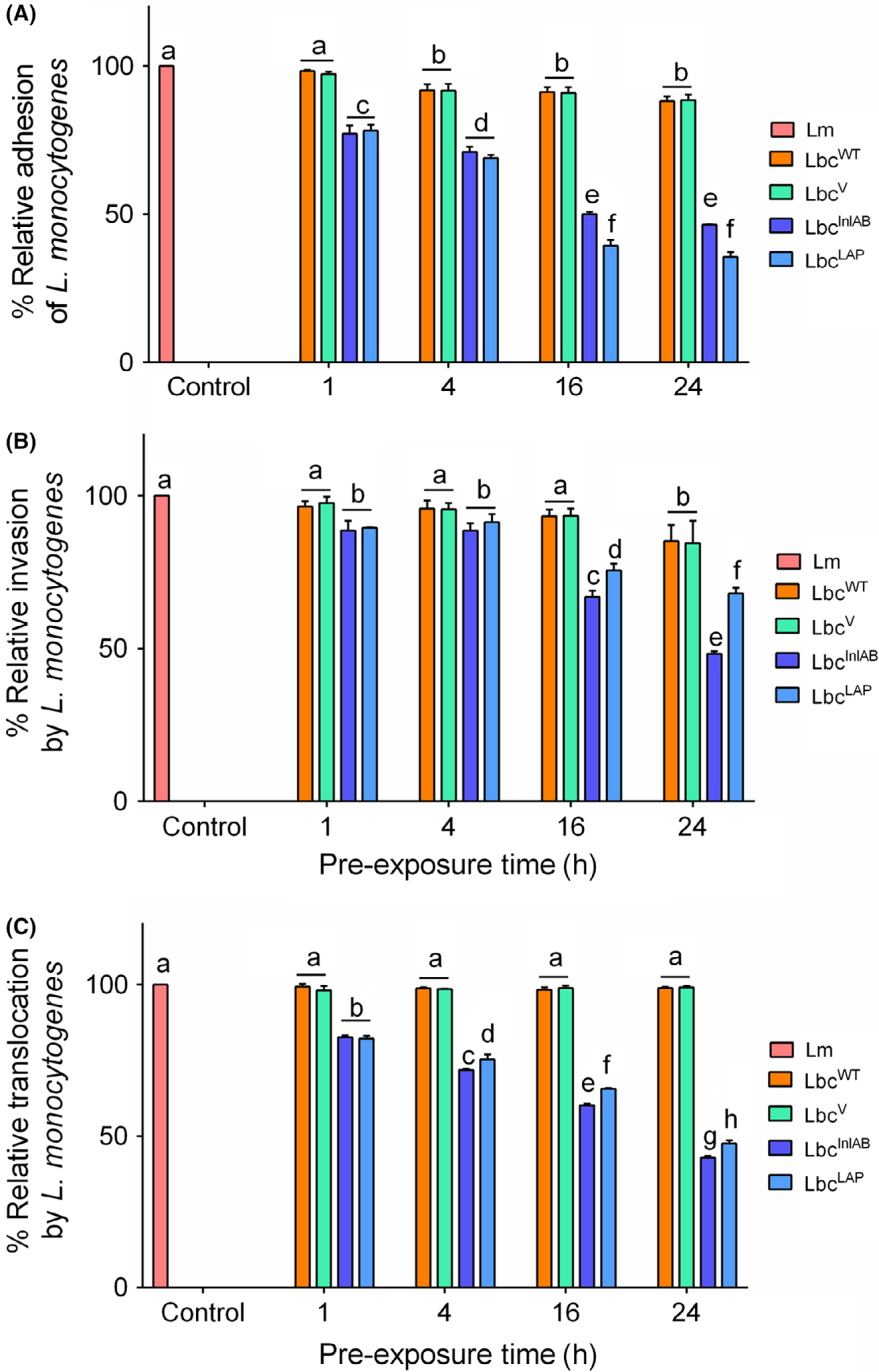
Inhibition of *Listeria monocytogenes* (Lm) adhesion (A), invasion (B) and transcellular translocation (C) by the *L. casei* strains (Lbc^WT^, Lbc^V^, Lbc^Inl^ ^AB^ and Lbc^LAP^). Caco‐2 cells were pre‐exposed to *L. casei* strains for 1, 4, 16 and 24 h before infection with Lm for 1 h for adhesion and invasion and 2 h for translocation. Data are averages of three experiments ran in duplicates. For each time point, bars marked with different letters (a, b, c, d, e, f, g, h) indicate significant difference at *P* < 0.05.

Next, we compared the inhibitory effect of Lbc^InlAB^ pre‐exposed to Caco‐2 cells for 1, 4, 16 and 24 h of duration against *L. monocytogenes* infection (adhesion, invasion and translocation) for 1 h. Overall, the results indicated that reduction of the interaction between *L*. *monocytogenes* and Caco‐2 cells increased with increasing pre‐exposure time of the Caco‐2 monolayer to Lbc^InlAB^, while it was not or was negligibly affected by prolonged exposure to either Lbc^WT^ or Lbc^V^ (Fig. 5).

In the adhesion assay, Lbc^InlAB^ reduced *L. monocytogenes* adhesion by 50–53.6% at 16 and 24 h, while Lbc^WT^ and Lbc^V^ reduced by only 8% (Fig. 5A). As a positive control, Lbc^LAP^ showed about 64.43% reduction in *L. monocytogenes* adhesion to Caco‐2 cells at 24 h, which is significantly higher (*P* < 0.0001) than that of the Lbc^InlAB^ strain (Fig. 5A).

In the invasion assay, Lbc^InlAB^ reduced *L. monocytogenes* invasion by 51.7% at 24 h, while Lbc^WT^ and Lbc^V^ reduced invasion by only 15%. As anticipated, Lbc^LAP^ showed about a 32% reduction in *L. monocytogenes* invasion to Caco‐2 cells at 24 h, which is significantly lower than Lbc^InlAB^ (Fig. 5B).

In the transcellular translocation assay, Lbc^InlAB^ reduced *L. monocytogenes* translocation by 57.14% at 24 h, while Lbc^WT^ and Lbc^V^ did not show any reduction at the same pre‐exposure period. As a positive control, Lbc^LAP^ showed about 52.46% reduction in *L. monocytogenes* translocation to Caco‐2 cells at 24 h, similar to Lbc^InlAB^ (*P* = 0.1595) (Fig. 5C). These results collectively indicate that InlAB‐expressing *L. casei* reduced *L. monocytogenes* adhesion, invasion and transcellular translocation in the Caco‐2 cell model showing a pronounced inhibitory effect after 16–24 h pre‐exposure.

### Inhibition of cytotoxic effects of *L. monocytogenes* on Caco‐2 cells by *L. casei*

**Figure 6:**
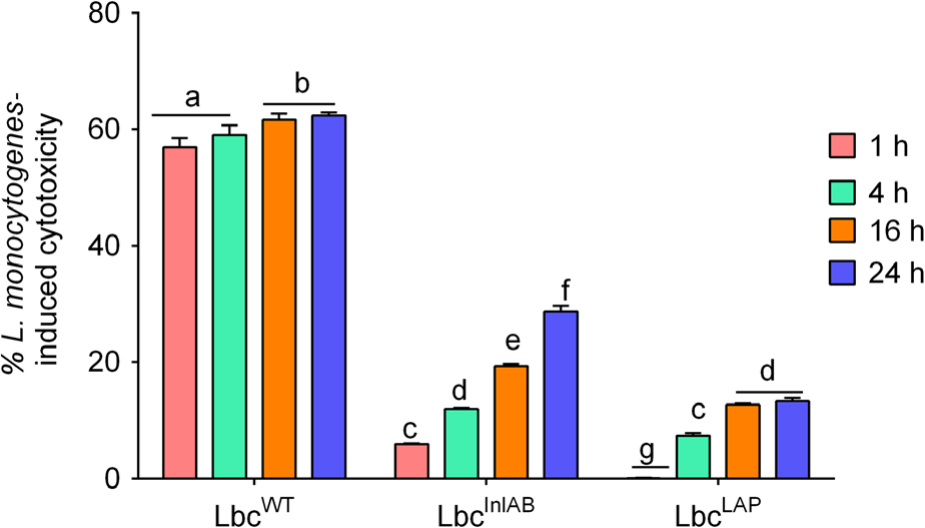
Cytotoxicity of *Listeria monocytogenes* in Caco‐2 cells pre‐exposed with *Lactobacillus casei* over time (1, 4, 16, 24 h). Cytotoxicity value for *L. monocytogenes* treatment (1 h) in the absence of *L. casei* strains was 64.38%. Data are averages of three experiments ran in duplicates. For each time point, bars marked with different letters (a, b, c, d, e, f, g) indicate significant difference at *P* < 0.05.

We investigated the cytotoxic effect by measuring lactate dehydrogenase (LDH) release induced by *L. monocytogenes* (1 h) from Caco‐2 cells in the presence or absence of *L. casei* strains (Fig. 6). *L. monocytogenes* treatment for 1 h induced 64.38% cytotoxicity to Caco‐2 cells in the absence of *L*. *casei*, while it induced only 5.93% and 28.7% cytotoxicity after 1 h and 24 h pre‐exposure to Lbc^InlAB^, respectively, and by 57% and 62.3% after 1 and 24 h pre‐exposure to Lbc^WT^ respectively (Fig. 6). Interestingly, *L. monocytogenes* induced only 0.09% and 13.3% cytotoxicity after 1 and 24 h pre‐exposure to Lbc^LAP^ respectively. Pre‐treatment of Caco‐2 cells with recombinant *L*. *casei* strains resulted in their significant protection (*P* < 0.0001) against the cytotoxic effect of *L. monocytogenes* compared to pre‐treatment with Lbc^WT^.

### Recombinant Lbc^InlAB^ protects epithelial tight junction barrier integrity

**Figure 7:**
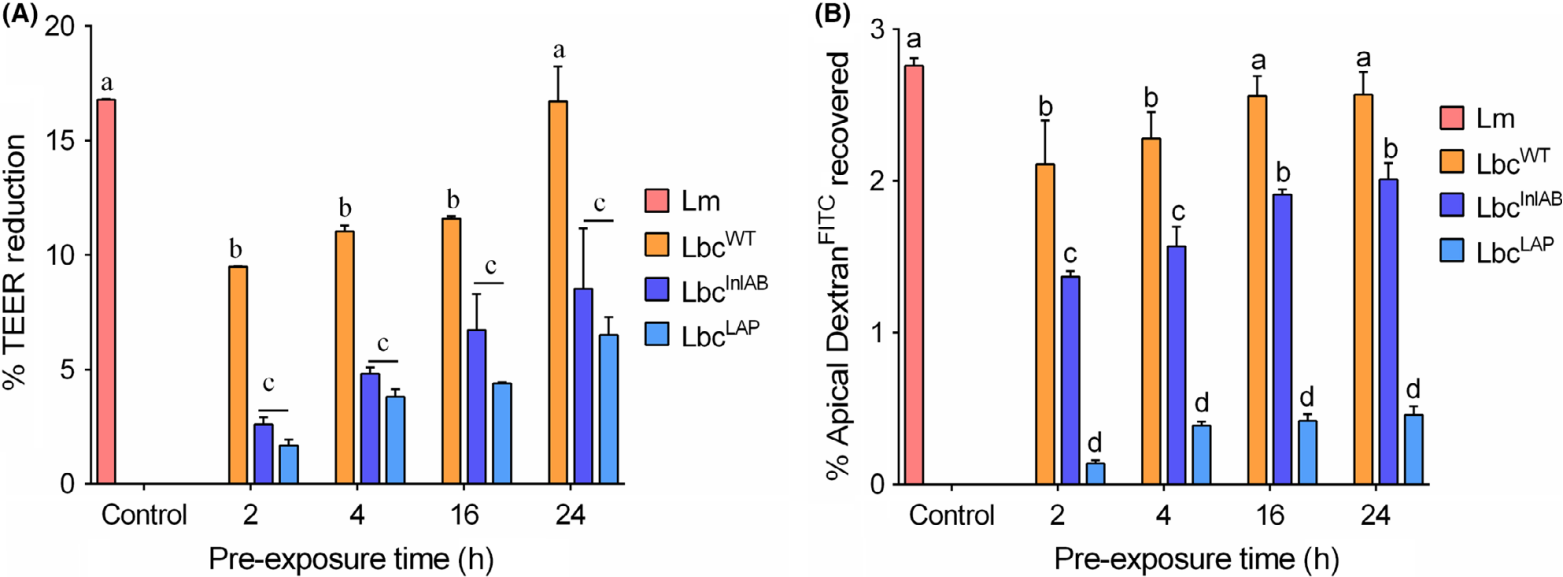
Caco‐2 cell permeability analysis using (A) transepithelial electrical resistance (TEER) and (B) 4 kDa of dextran^FITC^ (FD4) permeability assay. Caco‐2 cell monolayers were grown in trans‐well inserts and treated with *L. casei* strains (Lbc^WT^, Lbc^V^, Lbc^Inl^ ^AB^ or Lbc^LAP^) for 2, 4, 16 and 24 h, before their infection with *L. monocytogenes* (Lm) for 2 h. TEER measurements before and after exposure to *L. monocytogenes* treatment alone were 268.9 ± 2.3 and 224.5 ± 4.7, respectively, with a 16.5% change. Values are averages of two experiments analysed in triplicate. Per cent TEER reduction was calculated as per Koo *et al*. (Koo *et al*., 2012) as 1 – TEER_after_/TEER_before_ ×100.B. FD4 recovery after Lm was 2.76 ± 0.03%. Values are averages of three independent experiments performed in triplicates. For each time point, bars marked with different letters (a, b, c, d) indicate significant difference at *P* < 0.05.

We further monitored the effect of recombinant *L. casei* strains on *L*. *monocytogenes*‐mediated tight junction barrier function of Caco‐2 cells by measuring the transepithelial electrical resistance (TEER) and permeability of 4 kDa of dextran^FITC^ (FD4). The TEER value for Caco‐2 cells exposed to *L*. *monocytogenes* for 2 h without *L. casei* pre‐treatment was 16.9%. When Caco‐2 cells were pre‐exposed to *L. casei* strains, TEER values were between 9.5% and 16.7%, 2.6% and 8.53%, and 1.67% and 6.52% for Lbc^WT^, Lbc^InlAB^ and Lbc^LAP^ respectively (Fig. 7A). There was a significant (*P* < 0.0001) protection of epithelial barrier disruption by Lbc^InlAB^ and Lbc^LAP^ strains compared with Lbc^WT^. However, prolonged pre‐exposure (24 h) to all *L. casei* strains resulted in a decrease in TEER values for all the treatments.

We also measured the FD4 (paracellular marker) permeability through the epithelial barrier in a trans‐well set‐up to assess the epithelial barrier integrity. When the Caco‐2 cells were only infected with *L. monocytogenes* for 2 h, 2.76% of the FD4 was recovered at the basal side (Fig. 7B). The FD4 level decreased to 1.3% when Caco‐2 cells were pre‐exposed to Lbc^InlAB^, and 2.1% when pre‐exposed to Lbc^WT^ for 1 h. A similar trend was observed at 4, 16 and 24 h. As a positive control, Lbc^LAP^ showed the highest protection against *L. monocytogenes*‐mediated epithelial barrier disruption showing FD4 permeability of only 0.1–0.3%. Nevertheless, these results show that Lbc^InlAB^ can prevent epithelial barrier disruption from *L. monocytogenes* infection much greater than the Lbc^WT^.

## Discussion

Most pathogens initiate infection of their host through the interaction of specific receptors using adhesive molecules on their surfaces (Kline *et al*., 2009). Beneficial bacteria (probiotics) prevent pathogen colonization by virtue of occupying the host cell surface receptors (Ryan and Bhunia, 2017; Jayashree *et al*., 2018). Therefore, the development of strategies to prevent pathogen interaction with the host provides a logical and effective intervention step. This can be achieved through expression of the virulence genes coding for molecules that bind to host cell receptors, in probiotic bacteria (Steidler, 2003; Paton *et al*., 2010; Aguilar *et al*., 2011; Kajikawa *et al*., 2011; Amalaradjou and Bhunia, 2013; Wolfe *et al*., 2017).

*Listeria monocytogenes* is responsible for a fatal infection in immunocompromised population, and pathogenesis depends on its ability to adhere and invade host cells in the gastrointestinal tract (Nikitas *et al*., 2011; Drolia *et al*., 2018; Drolia and Bhunia, 2019). Hence, blocking of adhesion and invasion events would be a logical robust option for preventing *L. monocytogenes* infection through its targeted inactivation (Amalaradjou and Bhunia, 2013; O'Toole *et al*., 2017). InlA and InlB are considered major invasion proteins required for *L. monocytogenes* adhesion and invasion into host cells (Radoshevich and Cossart, 2018). In this study, we successfully expressed InlA and InlB into *L. casei* (Lbc^InlAB^) (Fig. 1) to prevent *L. monocytogenes* interaction with an intestinal cell line. Molecular weight of InlB in Lbc^InlAB^ in the cell wall fraction was found to be slightly higher (~80 kDa) than the actual MW in *L. monocytogenes* WT (Lm) (Fig. 1, Fig. S1) possibly because of coexpression of InlB (67 kDa) with the PrtP (PII‐type Proteinase) anchor with cell wall (117 aa = 12.87 kDa) (Maassen *et al*., 1999), while the MW of InlA remained the same in Lbc^InlAB^ because it possibly employed its own LPXTG motif to anchor the cell wall peptidoglycan (Bierne and Cossart, 2007). Often, the expression of new genes in a heterologous strain can result in changes in the growth and physiology of the recombinant strain (Ramos *et al*., 2004; Li *et al*., 2016). The growth rates of Lbc^InlAB^ and Lbc^WT^ were similar (Fig. 2), suggesting that expression of the extra genes by the recombinant *L*. *casei* did not affect its growth. This is a desirable outcome as it indicates that growth and potential consequent colonization of the recombinant would be comparable to those of the parental strain.

Expression of InlAB in Lbc^InlAB^ strain enhanced its ability to adhere, invade and translocate across the epithelial cell barrier. Increased adhesion of Lbc^InlAB^ to epithelial cells (Fig. 3) is highly desirable for its optimal functionality (Candela *et al*., 2008; Duary *et al*., 2011) and for creating a barrier for pathogen interaction with the host cells (Lee and Puong, 2002; Koo *et al*., 2012). Lbc^InlAB^ strain exhibited higher invasion and paracellular translocation through the epithelial barrier than Lbc^WT^ and Lbc^LAP^ (Fig. 3). The lower invasion by Lbc^LAP^ was expected since LAP is not involved in intracellular invasion (Burkholder and Bhunia, 2010). These findings, in part, corroborate with the previous studies where InlA‐expressing *L. lactis* was able to invade enterocytes efficiently (Guimaraes *et al*., 2005; Innocentin *et al*., 2009; De Azevedo *et al*., 2015) by transcytosis (Nikitas *et al*., 2011; Drolia and Bhunia, 2019). The major concern remains if such a strain could cross the epithelial barrier to spread systemically and consequently cause undesirable effects such as bacteraemia or septicaemia (Didari *et al*., 2014). *L. casei* is a widely used non‐pathogenic probiotic strain (Galdeano and Perdigon, 2006; Amalaradjou and Bhunia, 2012) lacking other virulence factors required for the systemic spread. It is thus anticipated that its recombinant strain expressing only InlAB will be cleared by immune cells rapidly from lamina propria. Previous studies indicated that some lactobacilli spontaneously translocate across the gut barrier; however, they were cleared within a short period by the host immune system, even when administered in higher dosages (Pavan *et al*., 2003; Liong, 2008). However, since these studies assessed natural infections using unmodified commensals, the safety of our recombinant strain will have to be tested and confirmed using *in vivo* experiments.

Others (Gueimonde *et al*., 2006; Collado *et al*., 2007) revealed that the degrees of probiotic strain adhesion and of its competitive adhesion, inhibition and/or displacement of the pathogen are not proportional. Therefore, adhesion of the probiotic should always be investigated simultaneously with its ability to reduce the adhesion of the pathogen to the same cells. Lee *et al*. (2003) reported that when incubated together, lactobacilli were able to compete with eight pathogens for adhesion to Caco‐2 cells*. *However, Collado *et al*. (2007) found that co‐incubation of probiotics and pathogens resulted in an increase in the adhesion of some pathogens. In our study, adhesion of *L*. *monocytogenes* to Caco‐2 cells was similar in the presence or absence of Lbc^WT^, indicating the limitation of this wild‐type strain to compete with this pathogen for the adhesion site on the cells (Fig. 4). Conversely, we found that both co‐incubation with and pre‐exposure to the recombinant Lbc^InlAB^ and Lbc^LAP^ significantly decreased *L. monocytogenes* adhesion, findings similar to previous reports (Lee and Puong, 2002; Jankowska *et al*., 2008; Koo *et al*., 2012). Furthermore, all *L. casei* strains were unable to displace *L. monocytogenes* already attached to Caco‐2 cell monolayer, similar to previously published studies (Lee *et al*., 2003; Candela *et al*., 2008; Koo *et al*., 2012). Our results suggest that the recombinant *L*. *casei* will be effective as a prophylactic rather than a therapeutic intervention.

Next, we examined whether prolonged exposure to *L*. *casei* strains would offer higher protection against *L. monocytogenes* infection. A 16–24 h pre‐exposure to Lbc^InlAB^ showed the highest anti‐listeria effect for all three stages of infection modalities: adhesion, invasion and translocation (Fig. 5). The anti‐adhesive and anti‐invasive activities of Lbc^InlAB^ can be explained by its preoccupation of E‐cadherin or c‐Met receptors, which prevents *L. monocytogenes* adhesion and invasion by physical hindrance. Likewise, reduction in *L. monocytogenes* transcellular translocation is the consequential result of inhibition of its adhesion to the host cell receptor by Lbc^InlAB^. Lbc^LAP^ also showed reduced *L. monocytogenes* translocation, which could be attributed to probiotic‐induced physical hindrance and maintenance of tight junction integrity thus preventing pathogen passage (Pagnini *et al*., 2010; Bron *et al*., 2017). Indeed, both Lbc^InlAB^ and Lbc^LAP^ were able to prevent *L. monocytogenes*‐mediated epithelial barrier dysfunction and helped maintain epithelial barrier integrity since dextran (paracellular marker) movement was significantly reduced in the Caco‐2 monolayer from apical to the basal compartment in the trans‐well set‐up (Fig. 7).

In conclusion, expression of key virulence genes by probiotic strains offers an alternative strategy with potential for targeted control of *L. monocytogenes* infection. LAP‐expressing probiotic provided protection against infection in our previous *in vitro* study (Koo *et al*., 2012). In this study, we also show that expression of InlAB by *L*. *casei* can also provide protection against infection *in vitro*. Therefore, recombinant *Lactobacillus* strains expressing different virulence genes of *L*. *monocytogenes* can be targeted at different stages of its infection cycle such as adhesion, invasion and translocation. These recombinant strains will be effective as a prophylactic rather than therapeutic intervention for pathogens and for conferring general health beneficial effects.

Although the findings reported in this paper for use of recombinant *L*. *casei* strain expressing inlA and inlB for control of *L*. *monocytogenes* infection are promising, additional *in vivo* studies are required to determine its suitability for direct application in humans. Such *in vivo* trials should determine the persistence of the recombinant strain, the stability of the plasmid and expression of foreign genes in the absence of antibiotic pressure and presence of glucose, demonstrate *L*. *monocytogenes* disease reduction and address safety issues relating to its applications.

## Experimental procedures

### Bacterial strains, plasmids and growth conditions

Bacterial strains and plasmids used in this study are listed in Table 1. *L. monocytogenes* F4244 (serovar 4b, clinical epidemic strain) was cultured in tryptone soy broth supplemented with 0.6% yeast extract (TSB‐YE) or brain heart infusion (BHI) broth at 37°C for 18 h. The vector pLP401‐T (Pouwels *et al*., 2001) containing the pAmy promoter was used for the expression of InlAB in *L. casei* ATCC344. *E. coli* DH5α with vector was grown in Luria–Bertani (LB) broth supplemented with 50 μg ml^−1^ ampicillin. Wild‐type *L. casei* (Lbc^WT^) was grown in de Man Rogosa and Sharpe (MRS) broth, while the *L*. *casei* carrying the pLP401T empty vector (Lbc^V^) and recombinant Lbc^InlAB^ and Lbc^LAP^ (unpublished) strains were grown anaerobically at 37°C for 16 h in MRS broth containing 2 μg ml^−1^ erythromycin. To induce expression of InlAB and LAP by recombinant *L. casei* strains, the recombinants were grown in modified MRS broth (1% w/v protease peptone, 0.5% w/v yeast extract, 0.2% w/v meat extract, 0.1% v/v Tween‐80, 37 mM C_2_H_3_NaO_2_, 0.8 mM MgSO_4_, 0.24 mM MnSO_4_, 8.8 mM C_6_H_14_N_2_O_7_ in 0.1 M potassium phosphate buffer, pH 7.0) supplemented with mannitol (1% w/v) (Koo *et al*., 2012) at 37°C for 16 h.

**Table 1:** Bacterial strains and plasmids

Bacterial/plasmids	Strains	Description	Source
*Listeria monocytogenes*	F4244	Wild type, serotype 4b, epidemic strain	Our collection
*Lactobacillus casei*	ATCC344	Wild type	ATCC
*Escherichia coli*	DH5α	Wild type	Our collection
*L. casei*	AKB904 (Lbc^LAP^)	*L. casei* expressing *Listeria* adhesion protein of *L. monocytogenes* F4244 (Em^R^ 2 μg ml^−1^)	Our laboratory
*L. casei*	AKB908 (Lbc^InlAB^)	*L. casei* expressing InlAB of *L. monocytogenes* F4244 (Em^R^ 2 μg ml^−1^)	This study
*L. casei*	AKB909 (Lbc^V^)	Vector control; *L. casei* carrying pLP401T plasmid without an insert (Em^R^ 2 μg ml^−1^)	This study
Plasmids			
pLP401T		*Lactobacillus* expression vector, (Am^R^ 50 μg ml^−1^ and Em^R^ 2 μg ml^−1^)	(Pouwels *et al*., 2001)
pLP401‐InlAB		pLP401T carrying *inlAB* of *L. monocytogenes* F4244	This study

### Construction of *Lactobacillus casei* harbouring Internalin A and B (inlAB) operons

The construction of the recombinant *L. casei* was done according to the methods described before (Maassen *et al*., 1999; Koo *et al*., 2012) with minor modifications. Briefly, chromosomal DNA of *L. monocytogenes* F4244 was extracted and *inlAB* operon was amplified with PCR using the primers: InlABExp‐F (NotI): TAGCGGCCGCAACTATTGAAAAAGGAGTGTATATAGTG and InlABExp‐R (XhoI): GTCTCGAGTTTCTGTGCCCTTAAATTAGC (Integrated DNA Technologies, Coralville, IA, USA) with an expected amplicon size of 4371 bp. The plasmid (pLP401T) containing the pAmy promoter was used for expression of *inlAB* in the probiotic *L. casei* ATCC344. InlA is anchored into *L*. *casei* through its own LPXTG motif while InlB has a GW motif, but it is possibly also fused to the C‐terminal region of PrtP containing an LPXTG motif (Fig. S1). The plasmid and the purified DNA were digested using the restriction enzymes NotI and XhoI (NEB) and subsequently ligated (T4 DNA ligase). The product of ligation was then designated pLP401T‐InlAB, which was used for electroporation into competent cells of *E. coli* and *L. casei* (Koo *et al*., 2012). Electroporated *E. coli* and *L. casei* cells were then incubated at 37°C for 1 h and 3 h respectively. Transformants harbouring pLP401T‐*inlAB* were subsequently selected on LB agar containing 50 μg ml^−1^ ampicillin and MRS agar containing 2 μg ml^−1^ erythromycin for *E. coli* and *L. casei* respectively. The plates were incubated at 37°C overnight for *E. coli* and 72 h for *L. casei*. Confirmation of the identity of *inlA* and *inlB* genes in Lbc^InlAB^ strain was done using PCR and sequencing.

### Analysis of InlAB expression by *L. casei*

The overnight (18 h) grown cultures of *L. monocytogenes*, Lbc^WT^, Lbc^V^ and Lbc^InlAB^ were centrifuged (7000 *g*, 10 min, 4°C), and proteins were harvested from the supernatant, cell wall and intracellular fractions as before (Burkholder *et al*., 2009; Koo *et al*., 2012). Equal amounts of proteins (10 μg) from each fraction were separated using SDS‐PAGE(7.5%). Protein bands were transferred to an Immobilon‐P membrane (Millipore, Billerica, MA, USA) and then immunoprobed with anti‐InlA antibody mAb‐2D12 (1.0 mg ml^−1^) (Mendonca *et al*., 2012) or anti‐InlB pAb‐404 (1:1000) (Lathrop *et al*., 2008) and reacted with horseradish peroxidase‐coupled anti‐mouse or anti‐rabbit secondary antibodies (Jackson Immuno Research, West Grove, PA) at 37°C for 1 h. The membranes were developed with an enhanced chemiluminescence kit (Thermo Fisher, Canoga Park, CA, USA).

Additionally, expression of InlA and InlB in the recombinant *L*. *casei* strains was determined by immunofluorescence staining. Overnight cultures were washed twice in PBS and incubated with the anti‐InlA mAb 2D12 and anti‐InlB pAb 404 (diluted 1:500 in PBS) at 37°C for 1 h. Subsequently, cells were treated with Alexa‐conjugated anti‐mouse IgG Fab2 Alexa Flour R555 and anti‐rabbit IgG Fab2 Alexa Flour R488 (Cell Signaling, Danvers, MA, USA) secondary antibodies diluted 1:500 in PBS and incubated in the dark at 37°C for 1 h. Between the treatments, cells were washed at least four times with 0.5% PBS‐Tween‐20. The cells were viewed under a fluorescence microscope (Leica, Wetzlar, Germany) equipped with SPOT Software version 4.6.4.2 (Diagnostic Instruments, Sterling Heights, MI, USA).

### Effect of InlAB expression on the growth of *L. casei*

The growth curve of Lbc^WT^, Lbc^V^ and Lbc^InlAB^ strains in MRS broth was conducted for 24 h by measuring the cell density (OD_600 nm_) in a spectrophotometer (Beckman DU80) and by plate counting. At each time point, the OD reading was taken, and culture (1 ml) was used for plating. Lbc^WT^ cells were counted on MRS agar, while those of Lbc^V^ and recombinant Lbc^InlAB^ were counted on MRS agar containing 2 μg ml^−1^ erythromycin grown anaerobically at 37°C for 48 h. This experiment was performed twice in triplicates. Additionally, the morphologies of overnight *L*. *casei* cultures were examined using phase‐contrast micrographs (Leica).

### Recombinant *L. casei* strain adhesion and invasion into Caco‐2 cells

*Caco‐2 cell culturing*: Human colon carcinoma cell line Caco‐2 (HTB37; American Type Culture Collection, Manassas, VA, USA) was cultured in Dulbecco's modified Eagle's medium (DMEM) with high glucose (HyClone™; GE, Logan, UT, USA) supplemented with 10% fetal bovine serum (FBS) (Atlanta Biologicals, Flowery Branch, GA, USA) (D10F). The cells were grown in flasks (Greiner Bio‐One) for up to 10–12 days or until differentiated and then trypsinized (Gaillard and Finlay, 1996). They were then seeded in 12‐well plates at a density of 1 × 10^5^ cells/well and incubated at 37°C in the presence of 7% CO_2_ for 10–12 days until they were differentiated and reached confluence (10^6^ cells/well).
*Adhesion and invasion assays*: Overnight (18 h) grown bacterial cultures were washed twice with PBS, adjusted to OD 600 = 1 and were suspended in D10F to a final concentration of 1 × 10^7 ^CFU ml^−1^ to achieve a multiplicity of infection (MOI) or multiplicity of exposure (MOE), 10. The Caco‐2 cell monolayer was washed three times using DMEM, and then exposed separately to the *L*. *casei* strains (Lbc^WT^, Lbc^V^, Lbc^InlAB^ or Lbc^LAP^) and *L. monocytogenes* and incubated for 1 h at 37°C in a gas atmosphere with 5% CO_2_ (Koo *et al*., 2012). To enumerate bacterial adhesion, the Caco‐2 cell monolayer was first washed thrice using DMEM and then treated with 0.1% Triton X‐100 (37°C, 10 min). For the invasion assay, the monolayers were exposed to *L. monocytogenes* and *L*. *casei* and then washed as performed in the adhesion assay, and treated with gentamicin (50 μg ml^−1^, 1 h) and with 0.1% Triton X‐100 (37°C, 10 min). The lysed cell suspensions from both adhesion and invasion experiments were serially diluted in PBS before plating on MRS, supplemented with erythromycin (2 μg ml^−1^) and modified Oxford (MOX) agar for Lbc^WT^, recombinant *L*. *casei* and *L. monocytogenes* respectively. All the plates were incubated at 37°C for 24–48 h before bacterial enumeration.

### Determination of *L. monocytogenes* exclusion mode by the recombinant *L. casei* strains

The competitive exclusion assay was performed as before (Koo *et al*., 2012) with minor modifications. Bacterial cultures were prepared as above and were suspended in D10F to a final concentration of 1 × 10^7 ^CFU ml^−1^. For competitive adhesion, *L. monocytogenes* was co‐inoculated with each of the *L*. *casei* strains (Lbc^WT^, Lbc^V^, Lbc^InlAB^ or Lbc^LAP^) to Caco‐2 cell monolayer (MOI, 10) and incubated for 1 h. Adherent bacteria were enumerated as above.

In the inhibition of adhesion assay, the Caco‐2 monolayers were first inoculated with each *L*. *casei* strain (MOE, 10) and incubated for 1 h, and washed to remove unbound bacteria using DMEM. *L. monocytogenes* was then added to the wells, and plates were incubated for 1 h, followed by an enumeration of adherent bacteria by plating. For displacement of adhesion, Caco‐2 cells were first inoculated with *L. monocytogenes* (MOI, 10) and incubated for 1 h, and washed to remove unbound bacteria. *L*. *casei* strains were then added to the wells, and plates were incubated for 1 h. Adhered bacteria were released by treatment with 0.1% Triton X‐100 (37°C, 10 min) and plated on MRS, supplemented with 2 μg ml^−1^ of erythromycin and MOX agar plates for enumeration of Lbc^WT^, recombinant *L*. *casei* and *L. monocytogenes* respectively.

### Inhibition of *L. monocytogenes* adhesion and invasion by *L. casei* strains

The Caco‐2 cell monolayers were washed and then exposed to the *L*. *casei* strains (MOE, 10) for 1, 4, 16 and 24 h at 37°C in the humidified incubator with 5% CO_2_. Excess medium in the wells containing unbound *L*. *casei* was removed and replaced with 500 μl of *L. monocytogenes* suspended in D10F (MOI, 10), and the plates were incubated for 1 h at 37°C with 5% CO_2_. The adherent bacteria were enumerated by plating as above.

For inhibition of *L. monocytogenes* invasion, the Caco‐2 cell monolayers were exposed to each *L*. *casei* strain (MOE, 10) for 1, 4, 16 and 24 h at 37°C with 5% CO_2_. Excess *L*. *casei* cells were removed and replaced with 500 μl of *L. monocytogenes* suspended in D10F (MOI, 10) and then incubated at 37°C with 5% CO_2_ for 1 h_._ The cell monolayers were washed, treated with gentamicin (50 μg ml^−1^) for 1 h and determined for invading bacteria by plating.

### Caco‐2 cell cytotoxicity

Caco‐2 cell cytotoxicity induced by *L. monocytogenes* after pre‐exposure to *L*. *casei* strains over time was determined by using the lactate dehydrogenase (LDH) release assay as previously described (Koo *et al*., 2012).

### Transcellular translocation of *L. casei* strains and subsequent inhibition of *L. monocytogenes* transepithelial translocation by recombinant *L. casei*

The Caco‐2 cells were grown in 12‐well trans‐well filter inserts (3 μm pore size) for 20–25 days to reach confluence (Burkholder and Bhunia, 2010; Drolia *et al*., 2018). Transepithelial electrical resistance of Caco‐2 cells was quantified using the Millicell ERS (Millipore), and a TEER value of more than 200 Ω cm^−2^ was used for all the experiments. For determining baseline translocation by *L*. *casei* strains or *L. monocytogenes*, the Caco‐2 cells were washed, and then, the bacteria were added (MOI, 10) separately to the apical side of the trans‐well at 37°C with 5% CO_2_ for 2 h. The liquid from the basal well was collected, serially diluted in PBS and then plated for the enumeration of bacterial cells (CFU ml^−1^).

For the inhibition of *L. monocytogenes* translocation*, L. casei* cells were first added to the apical wells (MOE, 10) and incubated for 1, 4, 16 and 24 h at 37°C with 5% CO_2_ and *L*. *casei* counts in the basal wells were determined by plating on MRS agar. Subsequently, excess *L*. *casei* cells were removed from the apical well, and replaced with 500 μl of *L. monocytogenes* (MOI, 10) and then incubated at 37°C with 5% CO_2_ for 2 h. *L. monocytogenes* counts in the basal wells were determined by plating on MOX agar plates.

### Epithelial tight junction integrity analysis

Quantification of TEER of Caco‐2 cells before and after the exposure to the bacteria was performed using a Millicell ERS system (Millipore) as described before (Burkholder and Bhunia, 2010). Furthermore, the integrity of the tight junctions between Caco‐2 cells was determined by measuring FD4 permeability in a spectrofluorometer (Burkholder and Bhunia, 2010; Koo *et al*., 2012).

### Statistical analysis

All data were analysed using Prism 7 software (GraphPad Software Inc., San Diego, CA, USA), and significance was assigned at *P* < 0.05. Where appropriate, Tukey's multiple comparisons test, with *P *<* *0.05 as a significant difference, was used to identify statistically significant differences.

## Conflict of interest

None declared.

## Ethical approval

This article does not contain any studies with human participants or animals performed by any of the authors.

## Supporting information


**Figure S1**. (A) Plasmid map (14.2 kb) of InlAB expression vector pLP401T (9.8 kb)‐InlAB (4.4 kb) (Pouwels *et al.*, 2001). Ery, erythromycin resistance gene; Amp, ampicillin resistance gene; Ori+ = origin of replication of *E. coli*, Ori‐ = origin of replication of *Lactobacillus*; InlAB, Internalin A and B; Pamy, a‐amylase promoter gene; ssAmy, secretion signal (36 aa) and the N‐terminus (26 aa) of a‐amylase gene; Anchor, cell wall anchor region (117 aa) of the *prtP* (PII‐type Proteinase) gene of *L. casei*; Tcbh, transcription terminator of the cbh (conjugated bile acid hydrolase) gene; Rep, repA gene. (B) Western blot showing expression of Internalin (InlA) and InlB in the recombinant *L. casei* strains (Lbc^InlAB−1^, Lbc^InlAB−2^, Lbc^InlAB−3^, Lbc^WT^ and Lbc^V^ in the different cellular fractions (supernatant, cell wall and intracellular) and *L. monocytogenes* F4244 (Lm). Molecular weight of InlB in Lbc^InlAB^ was slightly higher (~80 kDa) than the actual MW in *L. monocytogenes* WT (Lm) in the cell wall fraction possibly because of co‐expression of InlB (67 kDa) with the PrtP anchor (117 aa = 12.87 kDa) while the MW of InlA remained the same because it is using LPXTG motif to anchor the cell wall.

** **
