# Supplementary figures and images for: Lactobacillus casei expressing Internalins A and B reduces Listeria monocytogenes interaction with Caco‐2 cells in vitro
Source: Microb Biotechnol. 2019 Apr 15;12(4):715–29. doi: 10.1111/1751-7915.13407 (PMC6559204; doi:10.1111/1751-7915.13407)

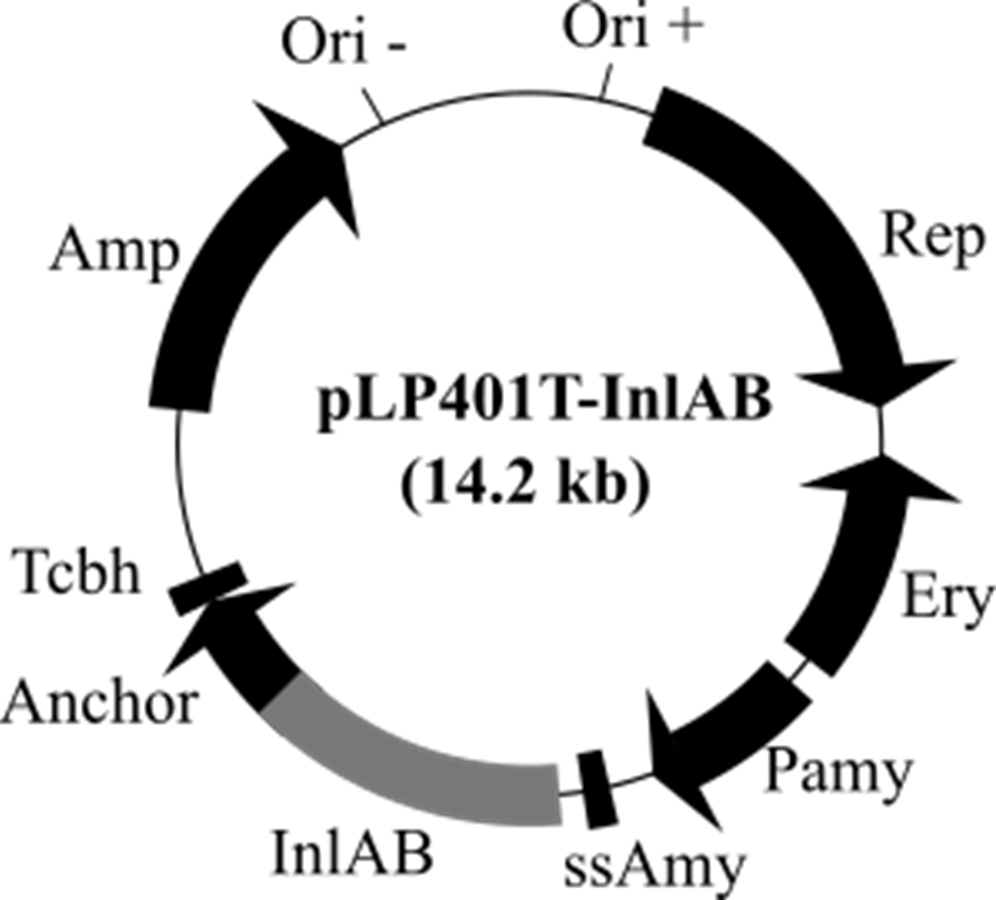

Supplement: Supplementary file 1 — Figure S1. (A) Plasmid map (14.2 kb) of InlAB expression vector pLP401T (9.8 kb)‐InlAB (4.4 kb) (Pouwels et al., 2001). Ery, erythromycin resistance gene; Amp, ampicillin resistance gene; Ori+ = origin of replication of E. coli, Ori‐ = origin of replication of Lactobacillus; InlAB, Internalin A and B; Pamy, a‐amylase promoter gene; ssAmy, secretion signal (36 aa) and the N‐terminus (26 aa) of a‐amylase gene; Anchor, cell wall anchor region (117 aa) of the prtP (PII‐type Proteinase) gene of L. casei; Tcbh, transcription terminator of the cbh (conjugated bile acid hydrolase) gene; Rep, repA gene. (B) Western blot showing expression of Internalin (InlA) and InlB in the recombinant L. casei strains (LbcInlAB−1, LbcInlAB−2, LbcInlAB−3, LbcWT and LbcV in the different cellular fractions (supernatant, cell wall and intracellular) and L. monocytogenes F4244 (Lm). Molecular weight of InlB in LbcInlAB was slightly higher (~80 kDa) than the actual MW in L. monocytogenes WT (Lm) in the cell wall fraction possibly because of co‐expression of InlB (67 kDa) with the PrtP anchor (117 aa = 12.87 kDa) while the MW of InlA remained the same because it is using LPXTG motif to anchor the cell wall. [file MBT2-12-715-s001.tif]

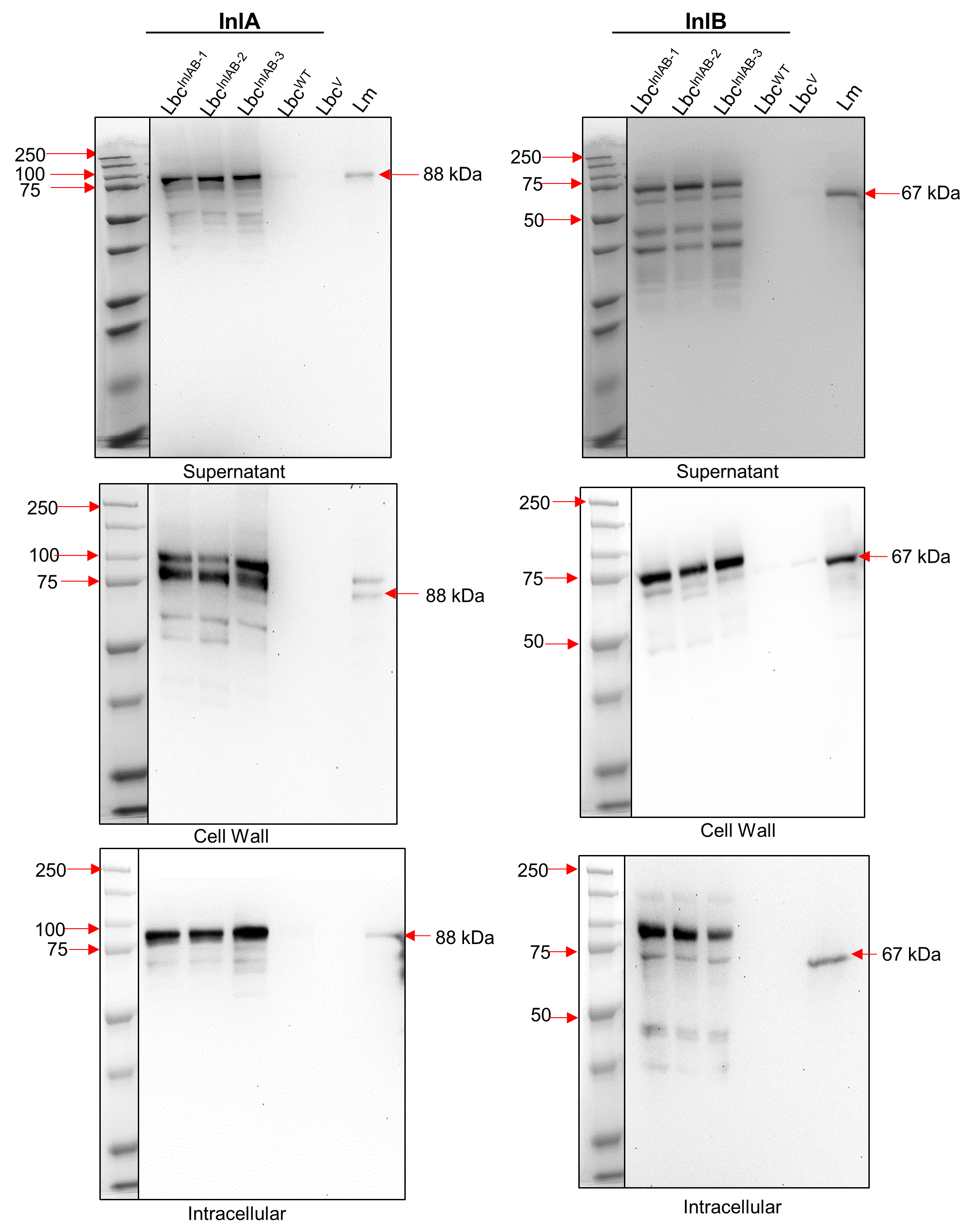

Supplement: Supplementary file 2 [file MBT2-12-715-s002.tif]
